# Impact of Treated Sewage Effluent on the Microbiology of a Small Brook Using Flow Cytometry as a Diagnostic Tool

**DOI:** 10.1007/s11270-015-2723-9

**Published:** 2016-01-22

**Authors:** Ibiye S. K. Harry, Ekwu Ameh, Frederic Coulon, Andreas Nocker

**Affiliations:** School of Energy, Environment and Agrifood, Cranfield University, Cranfield, MK43 0AL UK

**Keywords:** Microbiological water quality, Water ecology, Wastewater effluent, Flow cytometry, High nucleic acid bacteria, Low nucleic acid bacteria, Phosphate

## Abstract

Flow cytometry was applied to assess the microbiological impact of treated sewage effluent discharge into a small brook carrying surface runoff water. Increases in dissolved organic carbon and soluble reactive phosphorous were accompanied by increases in counts of intact bacteria by up to eightfold. Effluent ingress furthermore resulted in a pronounced shift of bacterial clusters. Whereas brook water upstream of the discharge point was characterised by a bacterial cluster with low nucleic acid (LNA) content, downstream water showed a shift to bacteria with high nucleic acid (HNA) content. Changes in the LNA/HNA ratio were largely maintained along the course of the brook. Results suggest that the LNA/HNA ratio can under certain conditions serve as an indicator of anthropogenic nutrient impact. Measuring impact on this low trophic level might be more sensitive and straightforward than measuring macroindicators. More evidence will however be required to assess the usefulness of LNA/HNA measurements to assess the ecological nutrient status of natural waters and the impact of nutrient pollution.

## Introduction

Nutrients are essential for the survival of the flora and fauna in any aquatic ecosystem with healthy waters being characterised by a balanced nutrient uptake and conversion stoichiometry (Dodds 2003). The ecological balance can be impacted by anthropogenic nutrient sources in cases where relatively high concentrations of nutrients are released into oligotrophic sensitive surface water bodies (Smith et al. 1999). Consequences can in some cases be severe and obvious (like algal blooms and fish kill due to hypoxia; Carpenter et al. 1998; White and Hammond 2006; Conley et al. 2009) and receive great public attention. In many cases, the impact however is moderate and not easily detected; thus, the monitoring strategy needs to be sensitive and requires careful choice. The ecological consequences on aquatic life are hereby traditionally measured by enumeration of water quality indicators like macroinvertebrates or algae (Jarvie et al. 2002) and on a microbiological scale by plate count enumeration methods of faecal indicator bacteria. The presence of the latter indicates the potential presence of sewage contamination. This applies however only in cases where discharged effluent has not undergone complete or partial disinfection.

In this study, we investigated the usefulness of flow cytometry (FCM) as a cultivation-independent analysis tool for measuring the impact of effluent discharge on water microbiology. A small brook flowing through the Cranfield University campus and receiving treated wastewater effluent from four relatively small sewage treatment works (STW, <10,000 populations equivalents) and runoff water from a number of small crop and livestock farm holdings in its catchment served as an example. FCM in its basic version can in principle measure two different things: firstly concentrations of total and intact bacteria and secondly (in contrast to traditional cultivation-based plate counting) distinct bacterial clusters can be visualised and quantified. Classifications of bacterial groups are exclusively based on staining properties using fluorescent nucleic acid stains. Whereas total cells can be detected with SYBR Green I (SG) that stains both intact and membrane-damaged cells, intact cells are identified by double staining with SG and propidium iodide (PI). The latter exclusively penetrates cells with damaged membranes resulting in a distinct shift in fluorescence (Berney et al. 2007). Both staining procedures (SG or SG/PI) allow the visualisation of cells with different nucleic acid content as first described for marine bacteria (Li et al. 1995; Marie et al. 1997). The two clusters have since then been identified in different aquatic systems (including brackish water, freshwater and drinking water; Lebaron et al. 2001; Bouvier et al. 2007; Van Wambeke et al. 2011) and are commonly referred to as low nucleic acid (LNA) and high nucleic acid (HNA) clusters (Lebaron et al. 2001). In the marine realm, LNA bacteria tend to comprise genome sizes of approx. <2 Mb, whereas HNA bacteria have genomes around 3–6 Mb (Schattenhofer et al. 2011).

In the light of tightening phosphate consents across Europe, a special emphasis was put on phosphate, which was identified as the most limiting nutrient in surface waters in the UK (Reynolds and Davies 2001; Mainstone and Parr 2002). Whereas 29 % of phosphorous in UK surface waters originates from diffuse agricultural sources (Defra 2010), approx. 60 % are introduced via treated domestic sewage effluent (White and Hammond 2006). Resulting eutrophication can cause loss of biodiversity (Carpenter et al. 1998; White and Hammond 2006) and increases mortality of fish and invertebrates (Wade et al. 2002). Elevated phosphate levels (in form of soluble reactive phosphate, SRP; Jarvie et al. 2002) in Chicheley brook had recently been observed by the Environment Agency with point sources as the likely contributors resulting in ‘bad phosphate status’ and ‘moderate ecological status’ classifications under the European Union Water Framework Directive (WFD) 2000/60/EC (Environment Agency 2014). The Cranfield University STW (permitted for up to 673 m^3^ day^−1^ of dry weather flow) was identified as a potential point source. SRP input that had already earlier been associated with a reduction in species diversity in the brook was indicated by an excess proportion of filamentous algae (*Cladophora* sp.) constituting around 25–50 % of the overall macrophytes (Dawson et al. 1999). This study aimed to investigate dissolved organic carbon (DOC) and phosphate levels along the brook and whether the impact of nutrient ingress would be reflected on a microbiological level by cultivation-independent FCM analysis.

## Materials and Methods

### Study Area

Samples were taken from Chicheley brook which is a watercourse that runs through a predominantly rural catchment including the villages of Cranfield, Hardmead, Chicheley, North Crawley, and Newport Pagnell. It is a tributary of the River Great Ouse and has a number of smaller streams and brooks discharging into it. Its national grid reference is SP94sw, and its water body ID is GB105033038040 (EA, 2014). Flow is largely dependent on rainfall. Land use in the catchment is predominantly small scale cropping, horticultural and livestock farm holdings including one dairy farm, two beef farms, two sheep farms, three grass-keeping farms and two large arable farms. Soil phosphorus (P) index in most of the farms ranges between 0 and 3 (low risk of P loss) with only two farms recording P index of 4 (medium P loss) in some of their fields. Two of the farms use treated sewage sludge supplied by a water company’s STW. Cranfield University STW collects and treats sewage effluent from Cranfield University STW before discharging into the brook.

### Sampling

**Fig. 1:**
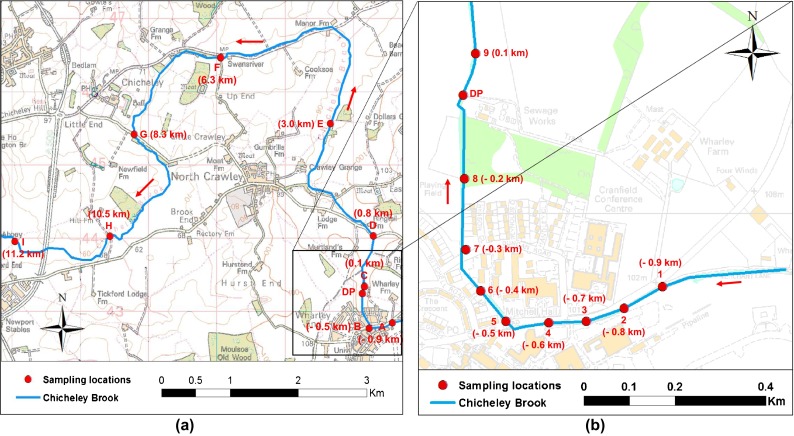
Sampling locations along Chicheley brook. **a** Locations along the entire course of the brook. **b** Locations for high resolution sampling along the brook within the Cranfield University campus area. *DP* indicates the treated effluent discharge point of the Cranfield STW. *Arrows* indicate the flow of the river

Nine sampling locations were defined along the entire course of the brook (Fig. 1a; locations A–I). These locations were supplemented by samples taken within the area belonging to the Cranfield University campus to obtain a spatially increased resolution for this area (Fig. 1b; locations 1–9). Sampling points A and B and 1–8 are located upstream of Cranfield University STW. Sampling points F, G and H were chosen to assess the impact of two smaller STWs (Hardmead STW and North Crawley STW) with points E and F being located upstream and downstream of Hardmead STW and points G and H located at the discharge point and downstream of North Crawley STW. Point I is located approx. 800 m upstream of the River Great Ouse with which Chichely brook combines.

Water sampling was carried out mid-summer 2014 starting late June and ending mid-August. During these periods, the weather was largely dry with little or no rainfall. Each set of samples for microbial and chemical analysis was typically collected within approx. 2–3 h using a stainless steel water sampler (the sampler was rinsed at each location before collecting samples) and transferred into 500-ml sterile plastic bottles (Aurora Scientific, Bristol, UK). Samples used for microbial growth assessment were collected in 1,000-ml Duran borosilicate glass bottles (Fisher Scientific Ltd., Loughborough, UK). All samples were transported in a cooler with ice and stored at 4 °C in the dark prior to analysis. Water for DOC analysis was transferred into glass bottles.

To assess the effect of seasonal variations on the impact of treated effluent discharge on microbial numbers in the brook, samples were collected in addition on a weekly basis over the time course of 12 months (July 2013 and July 2014) upstream of the discharge site (sampling point 8; Fig. 1), from the effluent itself and directly after the effluent had mixed with brook water. Samples were collected in borosilicate glass bottles and transported directly to the laboratory for microbiological analysis.

### Physical and Chemical Water Analysis

Water temperature was measured using a glass thermometer (Fisher Scientific Ltd, Loughborough, UK). Turbidity measurements were performed using a HACH model 2100N turbidity meter (Camlab, Cambridge, UK). The equipment was calibrated daily, and results were within 95 % accuracy. Water pH was measured using a VWR pH meter (VWR International Ltd., Leicestershire, UK). The equipment was calibrated before each batch of analysis. For DOC and SRP measurements, samples were filtered through a 0.45-μm Millex-HV syringe filter (Merck Millipore, Tullagreen, Ireland) prior to analysis. DOC analysis was carried out using a Shimadzu TOC-V analyser (Shimadzu, Tokyo, Japan). The equipment was calibrated with 100 mg L^−1^ standard solutions before each use and, in all cases, 100 % accuracy in the measurements was obtained. SRP measurements were carried out using Merck Millipore orthophosphate test kits with a measurement range of 0.05–5 mg L^−1^ using a Merck Spectroquant NOVA 60 spectrophotometer (Merck Millipore, Tullagreen, Ireland). The photometer was calibrated weekly, and analyses were performed in duplicates.

### Microbiological Analysis

Total and intact cell concentrations were measured using flow cytometry. Fluorescence staining was carried out following the protocol developed by Hammes et al. (2007, 2008) with a few amendments. Total cell concentrations (TCC) were obtained by staining with SG dye (cat. no S7567, Life Technologies Ltd., Paisley, UK), while intact cell concentrations (ICC) were obtained by staining with a mixture of SG and PI dye (cat. no. P3566, Life Technologies, Paisley, UK). The 10,000× stock of SG was diluted with dimethyl sulfoxide (DMSO; Fisher Scientific, Fair Lawn, NJ) to a working stock concentration of 100×). The dye mixture for ICC quantification consisted of five parts of 100× SG and one part of PI (1 mg mL^−1^, corresponding to 1.5 mM). For TCC and ICC measurements, 2 μL of 100× SG or 2.4 μL of SG/PI mixture was aliquoted into 96-well flat bottom plates (manufacturer), respectively, followed by addition of 200 μL of sample into each well using a multichannel pipette. Samples and dye were mixed thoroughly by pipetting up and down several times prior to incubation at 37 °C for 13 min in a Grant-bio PHMP thermo-shaker (Grant Instruments Ltd, Cambridgeshire, UK) at 600 rpm. The incubation time of 13 min corresponds to the standardised and validated method no. 333.1 for determining total cell counts and distinct bacterial clusters in freshwater as outlined in the Swiss Food Compendium (SLMB 2012). Final dye concentrations were 1× for SG and 3 μM for PI. Samples were analysed using a BD Accuri C6 cytometer (Becton Dickinson UK Ltd., Oxford, UK), at an excitation wavelength of 488 nm with all analyses carried out in triplicates. Samples with signals in excess of 10^6^ events mL^−1^ were diluted with 0.1-μm filtered (Millex-HV filter, Merck Millipore Ltd., Tullagreen, Ireland) Evian water (Evian, Evian-les-Bains, France). Green fluorescence by SG was collected in the FL1 channel (533 nm) and red fluorescence by PI in the FL3 channel (670 nm). TCC and ICC were analysed on an FL3 vs. FL1 density plot with trigger set on FL1, using the template and instrument settings described by Gatza et al. (2013). LNA and HNA clusters were quantified on the basis of a FL1 histogram as described earlier (SLMB 2012; Prest et al. 2013) with the difference that staining was performed with both SG and PI to limit detection to intact cells.

### Microbial Growth Potential Assessment

All glassware was made carbon-free using the method described by Charnock and Kjønnø (2000). The procedure comprised machine washing with phosphate-free detergent, followed by rinsing three times with ultrapure water (Milli-Q set at 18.2 Ω) and overnight acid wash using 0.2 M HCl. Acid was removed by rinsing three times with ultrapure water prior to air drying. Glassware was subsequently covered with aluminium foil and heated at 550 °C for 3 h using a muffle furnace (Carbolite, Derbyshire, UK). Water used for growth experiments was collected at sampling location B with 49 mL being transferred into 100-mL Duran borosilicate glass bottles (Fisher Scientific Ltd., Loughborough, UK). Samples were supplemented with either sodium phosphate or sodium acetate (1 mL each) to indicated final concentrations. Water without supplement (with 1 mL autoclaved ultrapure water being added alternatively) served as a control. Samples were incubated at 18 °C in an environmental test chamber providing light exposure (Sanyo, Moriguchi, Japan). To compare light with dark exposure, bottles were completely wrapped in aluminium foil in a repetition of the experiment to prevent light penetration. Experiments were performed in two independent repeats (consecutive samples) and microbial counts were measured at time point zero (*T*_0_) and after 7 days (*T*_7_) in triplicate. In addition to microbial counts, the algal density of the sample in each bottle after incubation (T7) was determined by measuring the chlorophyll a (chl. a) content of the sample. Chl. a extraction was performed according to the method described by Moed and Hallegraeff (1978). This comprised vacuum filtration of 49.5 mL of sample using a 1.2-μm Whatman GF/C glass microfiber filter (Fisher Scientific Ltd, Loughborough, UK) followed by boiling the filter with collected residue in 10 mL HPLC grade methanol (Fisher Scientific Ltd., Loughborough, UK) for 1 min to obtain the chl. a extract. The extract was thereafter centrifuged at 2,500 rpm for 3 min. A Jenway 6310 spectrophotometer (Camlab, Cambridgeshire, UK) was used to measure the absorbance of the extract at 665 and 750 nm using 10-mm light path cuvettes (Fisher Scientific Ltd, Loughborough, UK). Measurements were performed in duplicates, and the amount of Chl. a was estimated using the following equation:

$$ \upmu \mathrm{g}\ \mathrm{C}\mathrm{h}\mathrm{l}.\ \mathrm{a}\ {\mathrm{L}}^{-\mathsf{1}}=\frac{\mathsf{13.9}\ {\mathit{\mathsf{V}}}_{\mathrm{e}}\ \left(\mathrm{abs}\ \left(\mathsf{665}\right) - \mathrm{a}\mathrm{bs}\ \left(\mathsf{750}\right)\right)}{{\mathit{\mathsf{V}}}_{\mathrm{f}}\ \mathit{\mathsf{l}}} $$

where *V*_e_ = volume of methanol (mL), *V*_f_ volume of sample filtered (mL), *l* = length of light path (cm) and abs (665) and abs (750) = absorbance at 665 and 750 nm, respectively.

The phosphate stock solution (1000 mg L^−1^ PO_4_^3−^) was prepared by dissolving 183.25 mg of K_2_HPO_4_ in 100-mL ultrapure water. Similarly, acetate carbon solution (300 mg L^−1^ acetate carbon) was prepared by dissolving 102.5 mg of sodium acetate (C_2_H_3_NaO_2_) in 100 mL ultrapure water to give a 1.025-g L^−1^ acetate stock solution. Both stock solutions as well as 100 mL ultrapure water without supplement were autoclaved at 121°C for 15 min and stored at 4 °C for no more than 1 month. Dilute phosphate stock solutions were prepared in carbon-free glassware on the day of analysis using autoclaved ultrapure water.

### Statistical Analysis

The significance of LNA and HLA counts between upstream and downstream samples and effluent was determined by measures of analysis of variance (ANOVA with a confidence level of 95 %) followed by the post hoc Tukey’s multiple comparison test when *P* < 0.05. All tests were performed using GraphPad InStat version 3.0a (GraphPad Software, San Diego, CA, USA). Inter-correlated and right-skewed environmental variables (DOC, SRP, LNA, HNA) were square root transformed to stabilise the variance and identified using the Draftsman plot function of PRIMER v6 statistical software (Plymouth Marine Laboratory, Plymouth UK).

## Results

### Effect of Treated Effluent Discharge on Phosphorous, Carbon and Cell Concentrations

**Fig. 2:**
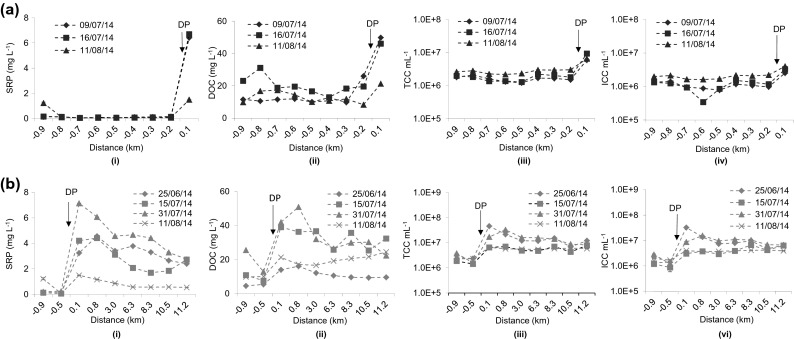
Spatial and temporal distribution of chemical and microbiological parameters along Chicheley brook **a** within the Cranfield University campus area and **b** along the entire course of Chicheley brook. *DP* = discharge point of Cranfield University STW-treated sewage effluent. (*i*) SRP, (*ii*) DOC, (*iii*) TCC mL^−1^, (*iv*) ICC mL^−1^

Chicheley brook was sampled on seven occasions; three of them included sampling within the Cranfield University campus area with spatially high resolution and four covering the entire course of the brook. All sampling events were performed during dry weather except for the last sampling (August 11, covering both categories), which took place within 24 h after rainfall. Concentrations of SRP and DOC are shown together with concentrations of total and intact cells in Fig. 2. The discharge of treated effluent resulted in a marked increase in SRP (in part above 6 mg L^−1^) and DOC (up to 50 mg L^−1^) compared to sample locations upstream of the discharge point (as seen both for the high resolution sampling within campus, Fig. 2a, and the extended brook sampling, Fig. 2b). To provide a context for SRP, concentrations should not exceed 0.12 mg L^−1^ for surface waters to attain a ‘good’ phosphorous status under the WFD classification (UK TAG 2008). The increase in SRP and DOC tended to be lowest when sampling was performed after rainfall indicating greater dilution of the effluent. Bacterial concentrations before effluent ingress tended to be slightly above 10^6^ cells mL^−1^ (which is a typical value for surface freshwater; Wang et al. 2007) and most cells were intact. Effluent-caused increases in total and intact cell concentrations were in the range between 2 to 7 and 1.8 to 8.3, respectively.

### Changes in Microbial Ecology

**Fig. 3:**
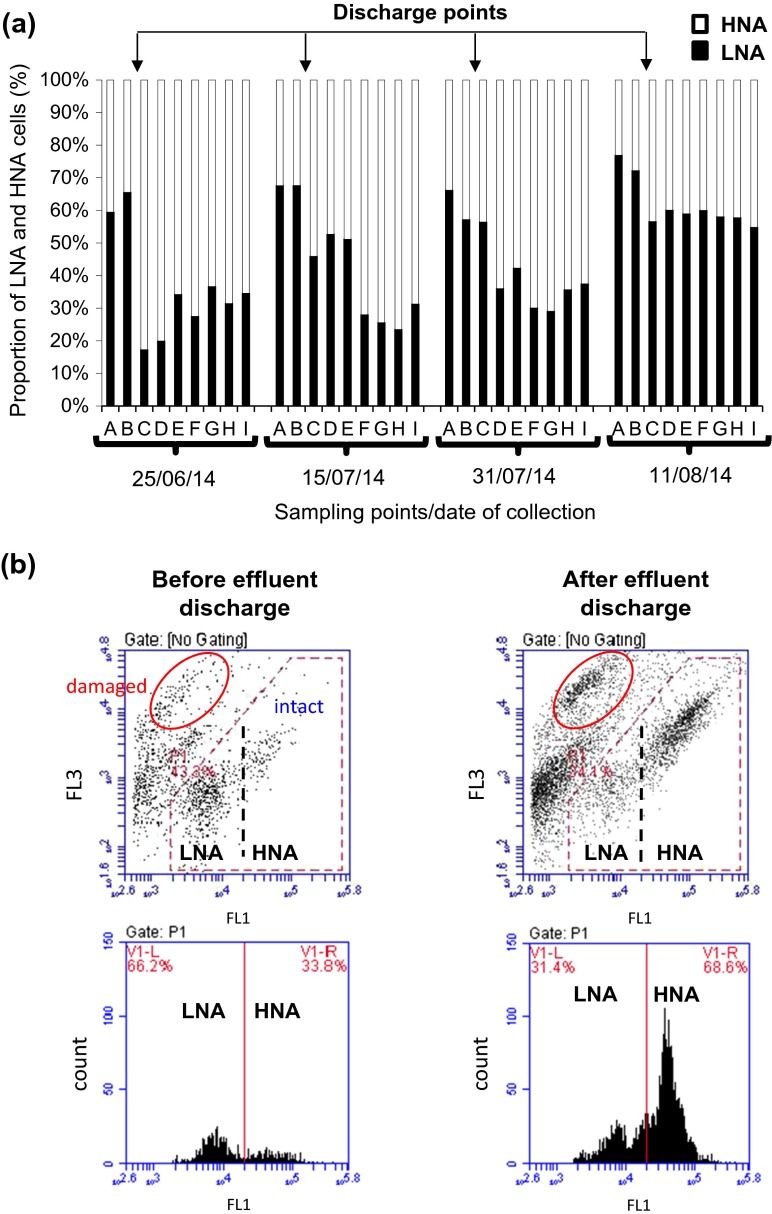
Impact of treated effluent discharge on water microbiology. **a** Proportion of LNA and HNA sub-clusters at sampling locations A to I along the entire course of Chicheley brook. **b** Representative FCM dot plots and histograms showing distribution of microbial clusters in brook water before and after receipt of treated effluent. Signals from intact cells are within the gated region (with LNA and HNA clusters indicated), and the position of damaged PI-positive cells is indicated by an *ellipse*. Other signals are caused by organic and inorganic background

Changes in the proportions between LNA and HNA before and after treated effluent discharge and along the brook are illustrated in Fig. 3a with a pronounced example of the shift in microbial clusters depicted in Fig. 3b. Whereas for samples upstream of the discharge point, the majority of detected bacterial cells belonged to the LNA cluster, the receipt of effluent resulted in a substantial shift of the LNA/HNA ratio towards elevated proportions of HNA bacteria. The weakest shift was obtained for the sampling event after rainfall as more dilution occurred. For all sampling events, high proportions of HNA bacteria (compared to upstream samples) were maintained along the course of the brook (approx. 11 km).

**Fig. 4:**
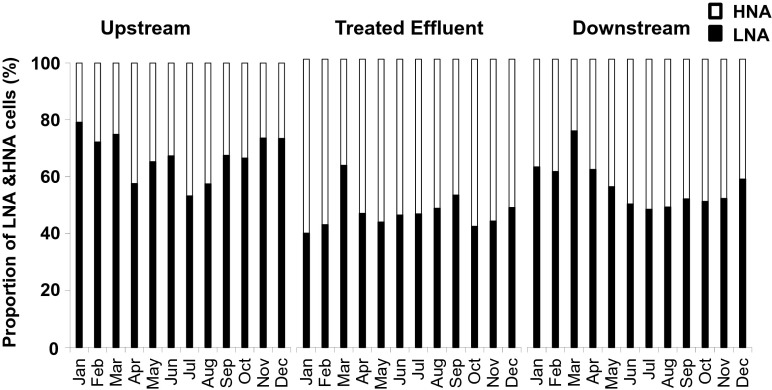
Monthly average proportions of LNA and HNA bacterial sub-clusters from locations upstream (sampling point 8) and directly downstream the treated effluent discharge point and the treated sewage effluent itself

When monitoring the bacterial clusters over the time course of 1 year, the proportion of LNA was consistently significantly higher for samples collected upstream of the discharge point with an average LNA proportion of 69 % compared to 57 % after receiving the treated effluent (ANOVA downstream vs. effluent *p* < 0.01). Conversely, HNA proportions were significantly higher after receiving treated effluent with an average of 43 % compared to 31 % in upstream samples. Average values for the discharged treated effluent itself were statistically different for LNA and HNA in comparison with upstream water (*p* > 0.001) and downstream water (*p* > 0.05). Changes of LNA/HNA ratios over 12 months are graphically shown in Fig. 4. A seasonal variation can be seen for upstream samples, where LNA proportions tended to be highest between September and March (average 72 %) compared to spring/summer (April to August, average 62 %). The trend was to a lesser extent still reflected in downstream samples, whereas the effluent itself seemed to reveal more stable proportions of LNA and HNA (with the March value being an outlier).

### Growth in Presence of Nutrients

Growth potential of bacteria contained in water upstream of the effluent discharge point (sampling point 2) was assessed by supplementing water with phosphate (final concentrations of supplement 1, 2 or 5 mg L^−1^), a carbon source (30 mg L^−1^ of acetate) or a mixture of those. Water without supplement served as a control. As shown in Table 1, numbers of intact cells increased within 7 days by factors between 1.6 and 6.8. Growth for the control sample without nutrient supplement was 3.8-fold and can potentially be attributed to assimilable organic carbon (AOC) that had been introduced into the brook prior the sampling event and not yet metabolised. Recent nutrient enrichment would explain why the sampled water had not reached its maximal level of biological stability (in the sense that water would not support further microbial growth; Hammes et al. 2010). Supplementing water with 30 mg L^−1^ carbon lowered growth to 1.6-fold, whereas highest growth (6.8-fold) was obtained after addition of 1 mg L^−1^ of phosphate. Higher concentrations of phosphate were associated with less growth both in absence and presence of carbon. Whereas these increases in microbial numbers were not dramatic and can probably be explained by limiting growth factors other than phosphate or carbon, strong changes in the ratios between bacterial LNA and HNA clusters were obtained. Whereas without nutrient supplement the proportion of HNA bacteria increased from 28 to 79 %, the increase was stronger in the presence of nutrients. Highest HNA proportions (89–92 %) were obtained when both phosphate and carbon were administered. The increase in HNA bacteria without added nutrient was confirmed in a repetition of the experiment in winter (December 2014; data not shown) to exclude a seasonal influence.

**Table 1:** Effect of added organic carbon and phosphate on microbial growth of intact cells numbers and proportions of LNA and HNA cells in comparison with a control sample without nutrient addition

	Phosphate supplement (mg L^−1^)	Microbial concentration						Regrowth factor
		*T*_0_			*T*_7_			
		ICC (mL^−1^)	LNA	HNA	ICC (mL^−1^)	LNA	HNA	
Control without nutrient supplement	n/a	96 × 10^5^ ± 2.8 × 10^4^	72 %	28 %	3.6 × 10^6^ ± 4.8 × 10^5^	21 %	79 %	×3.8
30 mg L^−1^	n/a				1.5 × 10^6^ ± 1.2 × 10^5^	11 %	89 %	×1.6
Phosphate	1				6.5 × 10^6^ ± 3.8 × 10^5^	12 %	88 %	×6.8
	2				3.9 × 10^6^ ± 1.2 × 10^6^	16 %	84 %	×4.1
	5				4.4 × 10^6^ ± 1.7 × 10^5^	13 %	87 %	×4.6
Phosphate + 30 mg L^−1^ carbon	1				3.3 × 10^6^ ± 1.8 × 10^4^	8 %	92 %	×3.4
	2				2.9 × 10^6^ ± 6.0 × 10^5^	11 %	89 %	×3.0
	5				1.9 × 10^6^ ± 4.9 × 10^5^	11 %	89 %	×2.0

Samples were measured at time zero (*T*_0_) and after 7 days (*T*_7_). Numbers present averages from two independent experiments (from consecutive samples) with all measurements performed in triplicate

### Correlations Between LNA/HNA, DOC and Phosphate

**Fig. 5:**
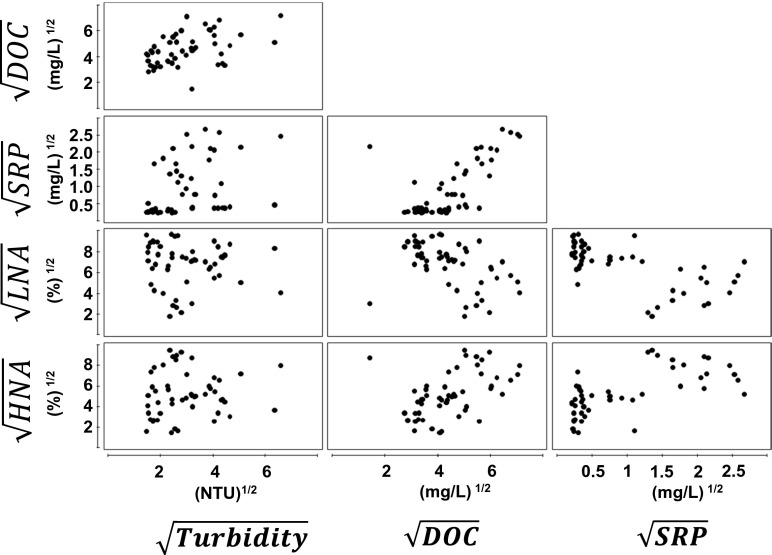
Draftsman scatter plots of square root-transformed values of HNA and LNA proportions with turbidity, DOC and SRP

The proportion of bacterial LNA and HNA clusters in environmental waters can be assumed to be influenced by a variety of factors of biological and physical nature. Correlating the proportions of these distinct clusters with selected parameters revealed a positive relationship between HNA bacteria with DOC, the inverse held true for LNA bacteria (Fig. 5). In regard to SRP, whose concentration is in relationship with DOC, the relationship is slightly different. For low concentrations of SRP up to approx. 1.5 mg L^−1^, the majority of bacteria belong to the LNA cluster (>50–77), whereas SRP concentrations >1.5 mg L^−1^ seem to shift the relative abundance to the HNA cluster. More data points will be necessary to corroborate whether there is a critical threshold in SRP concentrations.

## Discussion

We report here the impact of the discharge of treated sewage effluent on a brook carrying runoff water. The application of flow cytometry as a cultivation-independent diagnostic tool allowed not only quantifying bacterial numbers but also changes in distinct clusters (based on differences in cellular nucleic acid content and dye permeability). Both parameters were impacted by the effluent and remained changed over the studied course of the brook. Before effluent ingress, the majority of bacteria were classified as LNA similar to other freshwater bodies (Andrade et al. 2007; Belzile et al. 2008; Wang et al. 2009). LNA bacteria were reported to have a competitive advantage under nutrient limitation (Zubkov et al. 2001; Nishimura et al. 2005), although the studied brook has substantial DOC levels (Fig. 2) due to receipt of surface runoff water. Effluent discharge strongly shifted the ratio towards HNA bacteria tempting to speculate that the change in the nutrient concentrations were the cause. It is nevertheless not possible to conclude from our experiments how many of the HNA cells are introduced by the effluent itself and how many appear as a result of nutrient ingress and growth. The fact that an elevated HNA proportion was maintained for approx. 11 km (corresponding to the sampled course of the brook) indicates however a sustained effect.

The HNA status has historically been associated with metabolic activity, and bacteria belonging to this group were regarded as the ‘active part’ of the microbial community, whereas LNA bacteria were considered inactive or less active (Lebaron et al. 2001; Servais et al. 2003). This view was opposed by later studies reporting that also LNA bacteria contribute to metabolic activity both in seawater (Zubkov et al. 2001; Longnecker et al. 2005) and freshwater (Nishimura et al. 2005). Unquestionable evidence for activity of LNA bacteria was eventually provided by a study from Wang et al. (2009) reporting the isolation and cultivation of LNA bacteria from different freshwater systems. During growth, they maintained their small size and nucleic acid content. Growth was however limited to highly oligotrophic conditions with sterile freshwater as growth medium. Cell replication under high nutrient concentrations as given after effluent discharge is therefore likely to be limited to HNA cells, which subsequently outcompete LNA cells. Today, it is known that LNA and HNA bacteria are in part phylogenetically distinct and comprise different bacterial clades (Schattenhofer et al. 2011; Mary et al. 2006).

As environmental samples did not allow to conclude whether the increase in bacterial numbers and the shift in clusters was caused primarily by ingress of DOC, SRP or other factors, brook water from upstream the effluent discharge point was supplemented with phosphate or organic carbon. Both resulted in elevated HNA proportions. The strongest shift towards HNA was seen when both SRP and acetate were administered. In regard to SRP, this is in line with a study looking into variations of bacterial sub-clusters of a large mesotrophic lake in Japan (Nishimura et al. 2005). The percentage of cells with a very high nucleic acid content (VHNA) positively correlated with the concentration of dissolved inorganic phosphorous. VHNA growth was enhanced by addition of SRP or a combination of SRP and glucose but in contrast to our study not by the addition of a carbon source alone. Different nutrient limitations of the studied water would explain this deviation.

Surprisingly, water without supplement showed a strong shift from LNA to HNA (although not as strong as in the presence of added nutrients). Although enrichment with volatile AOC components by air exchange in the incubator cannot be excluded, water stagnation itself might be a possible reason as reported for tap water. Whereas for chlorinated tap water changes in microbial numbers and characteristics after dechlorination is easily explained with the loss of disinfectant residual (Lipphaus et al. 2014), also stagnation of non-chlorinated, biologically stable tap water was reported to lead to a substantial increase in the proportion of HNA cells (Lautenschlager et al. 2010). HNA cells in the latter study represented 48 ± 16 % of the bacterial community in flushed tap water with overnight stagnation raising the proportion to 66 ± 16 %. Residual AOC, higher water temperatures and nutrient leaking from water pipes in addition to household/network influences were discussed as possible reasons. Both residual AOC (e.g. by dead disintegrating biomass) and the change in temperature could play a role for the environmental samples studied here. Most importantly, LNA bacteria might be outcompeted by fast growing copiotrophic HNA bacteria due to a disruption of protozoan grazing. Although LNA bacteria are also subject to grazing, the grazing-related mortality tends to be much lower than the one of fast growing HNA bacteria in the presence of nutrients (Boenigk et al. 2004). Furthermore, in contrast to many other growth assays, our experiments were performed in the presence of light to assess a potential increase in photosynthetic algae. Although an increase in chlorophyll a concentrations could not be detected (data not shown), it cannot be excluded that metabolites from any given algae and nutrient competition might have influenced the bacterial community. Metabolic interactions between algae and bacteria have been reported previously (Cole 1982; Liu et al. 2012; Dittami et al. 2014). A repetition of the experiment in winter showed a stronger increase in HNA bacteria in the presence of light compared to dark incubation (data not shown).

As currently the meaning of different ratios of LNA and HNA bacteria remains unclear and no consistent correlation with certain environmental conditions has been described so far, more research will be necessary to verify whether HNA/LNA ratios may serve as indicators of anthropogenic nutrient input in waters with originally high LNA proportions. Monitoring at this low trophic level might however be an attractive and straightforward approach to measure the ecological consequences of changes in nutrient concentrations in waters that originally have a high proportion of LNA bacteria. It remains to be assessed with larger data sets whether relationships between bacterial clusters on the one hand and DOC and SRP on the other are different and how changes in water microbiology become reflected at higher trophic levels (e.g. using macroinvertebrates or algae) which are currently used as ecological indicators of surface water quality but which were not included in this study.

## Abbreviations

DOC: Dissolved organic carbon
FCM: Flow cytometry
HNA: High nucleic acid
LNA: Low nucleic acid
PI: Propidium iodide
SG: SYBR Green I
SRP: Soluble reactive phosphate
STW: Sewage treatment works
